# Paraneoplastische Rhabdomyolyse

**DOI:** 10.1007/s00108-025-01984-4

**Published:** 2025-09-05

**Authors:** Carlo Schneider, Patricia Faßbender, Linus Völker

**Affiliations:** 1 Klinik für Allgemeine Innere Medizin und Altersmedizin, Evangelisches Klinikum Köln Weyertal, Weyertal 76, 50931 Köln, Deutschland; 2 Abteilung für Gastroenterologie, Evangelisches Klinikum Köln Weyertal, Köln, Deutschland; 3 Klinik II für Innere Medizin – Nephrologie, Rheumatologie, Diabetologie und Allgemeine Innere Medizin, Uniklinik Köln, Universität zu Köln, Köln, Deutschland

**Keywords:** Myalgie, Hypokaliämie, Hyperaldosteronismus, Adrenokortikales Karzinom, Kasuistik, Myalgia, Hypokalemia, Hyperaldosteronism, Adrenal carcinoma, Case report

## Abstract

Eine 49-jährige Patientin stellte sich mit Verschlechterung des Allgemeinzustands und Muskelschmerzen vor. Laborchemisch imponierte die Konstellation einer Rhabdomyolyse bei ausgeprägter Hypokaliämie. Unter Kaliumsubstitution besserten sich die Beschwerden. Als Ursache der Hypokaliämie konnte ein Hyperaldosteronismus identifiziert werden. Sonographisch war eine Nebennierenraumforderung nachweisbar, die nach operativer Entfernung als Nebennierenkarzinom diagnostiziert wurde. Die Hypokaliämie ist eine seltene Ursache einer Rhabdomyolyse, die an einen Hyperaldosteronismus denken lassen sollte.

## Abstract (en)

A 49-year-old female patient presented with decline of general health and muscle pain. Laboratory findings showed a combination of rhabdomyolysis and severe hypokalemia. Symptoms improved with potassium supplementation. Hyperaldosteronism was identified as the underlying cause of the hypokalemia. Sonography revealed an adrenal mass that was diagnosed as adrenal carcinoma following surgical removal. Hypokalemia is a rare cause of rhabdomyolysis and should prompt consideration of hyperaldosteronism.

## Anamnese

Eine 49-jährige Patientin stellte sich mit Verschlechterung des Allgemeinzustands über 1 Woche, subjektiver Muskelschwäche und progredienten Muskelschmerzen der Extremitäten, beginnend mit Schmerzen in beiden Waden, in unserer Notaufnahme vor. Der Urin sei dunkel. Am Vortag sei durch die Hausärztin bereits Labordiagnostik erfolgt, die eine erhöhte Kreatinkinase (CK) sowie erhöhte Transaminasen gezeigt hätte. Vierzehn Tage zuvor sei es erstmals nach einer längeren Autofahrt zu Schmerzen in beiden Waden gekommen, die nach 2 Tagen wieder sistiert hätten. Vier Monate zuvor sei im Rahmen von Labordiagnostik eine Thrombozytopenie von 66/nl aufgefallen. Im Labor vom Vortag hätte sich weiterhin eine milde Thrombozytopenie (114/nl) gezeigt.

Relevante Vorerkrankungen seien nicht bekannt. Es sei zu keiner ungewohnten oder vermehrten körperlichen Belastung in der letzten Zeit gekommen. Es würden keine Medikamente eingenommen, insbesondere keine Statine. Drogenkonsum wird verneint. Es bestünden keine Infektzeichen, insbesondere kein Fieber. Auch Nachtschweiß, Gewichtsverlust und Gelenkschmerzen/-schwellungen werden verneint, ebenso (schläfenbetonte) Kopfschmerzen.

## Untersuchung

Es präsentierte sich eine Patientin in gutem Allgemeinzustand, mit unauffälligem körperlichem Untersuchungsbefund, insbesondere mit unauffälligem Kraftgrad der Extremitäten in der neurologischen Untersuchung. Der Blutdruck betrug in der Messung nach Riva-Rocci 146/90 mmHg, der Puls 65 Schläge in der Minute. Das Elektrokardiogramm (EKG) zeigte flache, fast isoelektrische T‑Wellen, war ansonsten mit einem normofrequenten Sinusrhythmus unauffällig.

## Diagnostik

Im Labor zeigten sich eine CK-Erhöhung von 10.180 U/l (Norm < 170) und eine Hypokaliämie mit 2,4 mmol/l (Norm 3,5–5,5). Ferner war eine milde Thrombozytopenie nachweisbar mit 123/nl (Norm 140–400). Die Laktathydrogenase (LDH) betrug 526 U/l (Norm < 250), die Aspartat-Aminotransferase (AST) 343 U/l (Norm < 35), die Alanin-Aminotransferase (ALT) 257 U/l (Norm < 35). Die Retentionsparameter waren normwertig. Die CK-MB war gemessen an der CK mit 87 U/l minimal erhöht (Norm < 35). Es war keine sog. Makro-CK nachweisbar. Thyrotropin (TSH) und Blutglukose waren im Normbereich. In der venösen Blutgasanalyse war der pH-Wert alkalisch mit 7,51 und die Basenabweichung lag bei 14 mmol/l (Norm 0–2). Das Standardbikarbonat war erhöht (40,6 mmol/l, Norm 22–26), bei einem Kohlendioxidpartialdruck (pCO_2_) von 52 mmHg.

## Therapie und Verlauf

**Abb. 1:**
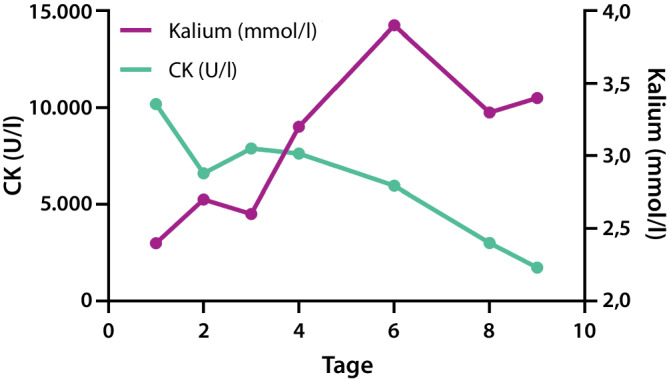
Darstellung der Spiegel der CK und des Serumkaliums während des Aufenthalts

Wir diagnostizierten eine Rhabdomyolyse und begannen eine parenterale Volumensubstitution und substituierten Kalium oral und intravenös. Darunter war die CK regredient, der Kaliumspiegel stieg an, persistierte jedoch unterhalb des Normbereichs (vgl. Abb. 1). Die metabolische Alkalose sahen wir im Zusammenhang mit der Hypokaliämie. Da im Rahmen der Rhabdomyolyse eher eine Hyperkaliämie zu erwarten gewesen wäre, stellten wir die Verdachtsdiagnose einer hypokaliämischen Rhabdomyolyse. In der hormonellen Diagnostik konnte ein erhöhter Aldosteronspiegel im Liegen (509 pg/ml, Norm < 232) bei supprimiertem Renin (< 1,0 µIU/ml, Norm 2,9–46,1) festgestellt werden. Unter Kochsalzbelastung ließ sich der Aldosteronwert nicht adäquat senken. Bei einem Renin-Aldosteron-Quotienten > 500 ergab sich die Konstellation eines primären Hyperaldosteronismus. Im Tagesprofil zeigten sich lediglich moderat erhöhte Blutdruckwerte.

**Abb. 2:**
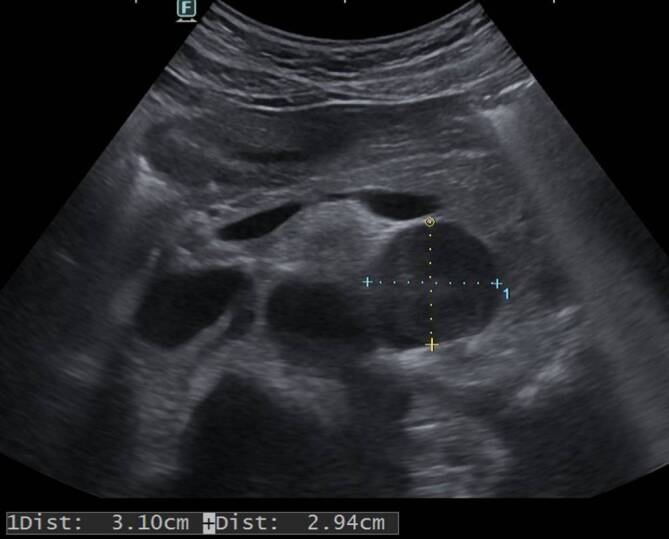
Sonographischer Transversalschnitt des Oberbauchs mit Darstellung einer ca. 3 × 3 cm großen inhomogenen Raumforderung paraaortal links

Wir führten eine Sonographie des Abdomens durch, in welcher sich keine hepatischen Auffälligkeiten zeigten. Jedoch stellte sich eine inhomogene, glatt begrenzte, paraaortale Raumforderung dar (vgl. Abb. 2). Ergänzend erfolgte eine Magnetresonanztomographie des Oberbauchs inklusive Nebennierenphase. Hier konnte die Raumforderung der linken Nebenniere zugeordnet werden. Der etwa 5 cm große Tumor war bildmorphologisch vereinbar mit einem hormonell aktiven Nebennierenadenom. Sonstige abdominelle Raumforderungen wurden nicht dargestellt.

Einen Hyperkortisolismus sowie eine erhöhte (Nor‑)Metanephrinproduktion konnten wir laborchemisch ausschließen. Aufgrund der Thrombozytopenie stand differenzialdiagnostisch initial auch eine Myositis im Rahmen einer rheumatologischen Erkrankung im Raum. C-reaktives Protein (CRP) und Blutsenkungsgeschwindigkeit (BSG) waren jedoch normwertig.

Nach Abschluss der hormonellen Diagnostik etablierten wir eine Therapie mit Spironolacton, unter welcher der Kaliumspiegel zuletzt nur noch leicht erniedrigt und die Blutdruckwerte normotensiv waren.

Nach Entlassung erfolgte im weiteren Verlauf die Tumorexstirpation. Überraschenderweise ergab sich histologisch der Nachweis eines adrenokortikalen Karzinoms, das R0-reseziert worden war. Die Patientin ist seither unter adjuvanter Behandlung mit Mitotan.

## Diskussion

Rhabdomyolysen kommen nicht selten vor und werden insbesondere nach ungewohnter körperlicher Belastung, Traumen oder im Rahmen von Drogen- und Medikamententoxizität beobachtet. Ebenso können angeborene Myopathien, endokrine Störungen und Elektrolytentgleisungen Rhabdomyolysen verursachen [1]. Eine seltene Ursache von Rhabdomyolysen ist die schwere Hypokaliämie [2], die z. B. als Folge eines primären Hyperaldosteronismus [3] oder auch im Rahmen von Lakritzeabusus [4] vorbeschrieben wurde. Aldosteron führt über eine erhöhte Anzahl von Natriumkanälen in den Verbindungstubuli und Sammelrohren der Niere zu einer gesteigerten Natriumrückresorption. In der Folge steigen typischerweise Blutvolumen und Blutdruck. Ferner führt der Kationenverlust im Harn zu einem elektrochemischen Gradienten, der den Kaliumstrom aus den Tubuluszellen in den Harn begünstigt und somit einen renalen Kaliumverlust antreibt [5]. Die mögliche Folge eines Hyperaldosteronismus ist daher die Kombination aus arterieller Hypertonie und Hypokaliämie. Nicht nur wird Kalium für die intrazelluläre Homöostase benötigt, interstitielles Kalium scheint die Perfusion von Muskeln unter Belastung zu regulieren, was das Auftreten von Muskelnekrosen im Rahmen ausgeprägter Hypokaliämien erklären könnte [6]. Adrenokortikale Karzinome sind eine seltene Entität, die insbesondere Kinder betrifft. Im Erwachsenenalter werden sie vor allem zwischen dem 30. und 50. Lebensjahr und häufiger bei Frauen diagnostiziert. Auch wenn ein Großteil der adrenokortikalen Karzinome hormonsezernierend ist, tritt ein Mineralokortikoidexzess nur in etwa 10 % der Fälle auf [7]. Paraneoplastische Rhabdomyolysen wurden schon vor über hundert Jahren unter dem Bild einer Polymyositis beschrieben. Üblicherweise liegen in diesem Zusammenhang jedoch autoimmunvermittelte Muskelnekrosen vor [8], die eher mit einer Hyperkaliämie einhergehen würden.

In dem hier vorliegenden Fall konnten durch die Anamnese bereits Medikamentennebenwirkungen und eine Trainingsüberlastung als Ursache der Rhabdomyolyse ausgeschlossen werden. Bei unauffälliger Familienanamnese und angesichts des Patientenalters war eine genetische Myopathie unwahrscheinlich. Durch das Aufnahmelabor schieden eine Hyper- oder Hypothyreose und eine diabetische Stoffwechsellage als Ursache der Rhabdomyolyse aus. Die Thrombozytopenie ließ uns an eine Myositis aus dem rheumatischen Formenkreis denken, der Verdacht ließ sich aber laborchemisch nicht erhärten. Trotz des nur grenzwertig erhöhten Blutdrucks wies die Hypokaliämie auf einen Hyperaldosteronismus hin. Dass in der Sonographie rasch eine Raumforderung gefunden werden konnte, bestärkte uns in unserer Arbeitsdiagnose. Einen Hyperkortisolismus und – da auch an ein Paraganglion gedacht wurde – eine erhöhte (Nor‑)Metanephrinproduktion schlossen wir aus. Aufgrund der eindeutigen Konstellation eines Hyperaldosteronismus und einer vergleichsweise großen Raumforderung verzichteten wir auf eine selektive Venenblutentnahme.

## Fazit für die Praxis

Eine Hypokaliämie sollte im Rahmen einer Rhabdomyolyse aufhorchen lassen, da diese mit hoher Wahrscheinlichkeit die Rhabdomyolyse antreibt und auf die Fährte der zugrunde liegenden Ursache weist.
